# APOBEC3 Proteins: From Antiviral Immunity to Oncogenic Drivers in HPV-Positive Cancers

**DOI:** 10.3390/v17030436

**Published:** 2025-03-18

**Authors:** Eliza Pizarro Castilha, Rosalba Biondo, Kleber Paiva Trugilo, Giulia Mariane Fortunato, Timothy Robert Fenton, Karen Brajão de Oliveira

**Affiliations:** 1 Laboratory of Molecular Genetics and Immunology, Department of Immunology, Parasitology and General Pathology, Center of Biological Sciences, State University of Londrina, Londrina 86057-970, Brazil; eliza.pizarro.castilha@uel.br (E.P.C.); klebertrugilo01@gmail.com (K.P.T.); giulia.fortunato@uel.br (G.M.F.); 2 Leiden Academic Centre for Drug Research, Analytical Biosciences, Leiden University, P.O. Box 9502, 2311 EZ Leiden, The Netherlands; r.biondo@lacdr.leidenuniv.nl; 3 School of Cancer Sciences, Faculty of Medicine, University of Southampton, University Hospital Southampton, Tremona Road, Southampton SO16 6YD, UK; t.r.fenton@soton.ac.uk; 4 Institute for Life Sciences, University of Southampton, University Road, Southampton SO17 1BJ, UK; 5 Polymorphism Research Laboratory, Department of Immunology, Parasitology and General Pathology, Center of Biological Sciences, State University of Londrina, Londrina 86057-970, Brazil

**Keywords:** deamination, APOBEC, cancer, HPV

## Abstract

The human APOBEC superfamily consists of eleven cytidine deaminase enzymes. Among them, APOBEC3 enzymes play a dual role in antiviral immunity and cancer development. APOBEC3 enzymes, including APOBEC3A (A3A) and APOBEC3B (A3B), induce mutations in viral DNA, effectively inhibiting viral replication but also promoting somatic mutations in the host genome, contributing to cancer development. A3A and A3B are linked to mutational signatures in over 50% of human cancers, with A3A being a potent mutagen. A3B, one of the first APOBEC3 enzymes linked to carcinogenesis, plays a significant role in HPV-associated cancers by driving somatic mutagenesis and tumor progression. The A3A_B deletion polymorphism results in a hybrid A3A_B gene, leading to increased A3A expression and enhanced mutagenic potential. Such polymorphism has been linked to an elevated risk of certain cancers, particularly in populations where it is more prevalent. This review explores the molecular mechanisms of APOBEC3 proteins, highlighting their dual roles in antiviral defense and tumorigenesis. We also discuss the clinical implications of genetic variants, such as the A3A_B polymorphism, mainly in HPV infection and associated cancers, providing a comprehensive understanding of their contributions to both viral restriction and cancer development.

## 1. Introduction

The apolipoprotein B mRNA-editing enzyme catalytic polypeptide-like (APOBEC) superfamily consists of a group of cytidine deaminase enzymes that play fundamental roles in immunity, metabolism, and the regulation of gene expression. These enzymes catalyze the conversion of cytidines into uridines in single-stranded DNA or RNA, a critical modification for various biological processes, such as antibody diversification in B cells, RNA editing for protein formation, and viral replication restriction [1].

The human APOBEC protein family comprises 11 members; among them, the APOBEC3 group has received special attention due to its role in innate immune defense against viruses and the control of mobile genetic element replication. Proteins such as APOBEC3G and APOBEC3B are essential in neutralizing viruses, including human immunodeficiency virus (HIV) and human papillomavirus (HPV), through the induction of mutations in viral DNA, thereby inactivating the pathogen’s genetic material [2]. However, these activities are also associated with adverse effects, such as the introduction of somatic mutations into the cellular genome, a phenomenon that may contribute to genomic instability and tumor progression [3].

Over the past 10 years, numerous studies have linked the mutagenic activities of APOBEC3A and APOBEC3B to the generation of mutational signatures observed in over 50% of human cancers [3,4,5]. Mutagenesis induced by these enzymes occurs predominantly during single-stranded DNA states, such as those seen during replication and transcription, and is exacerbated under conditions of replicative stress [6]. Additionally, the presence of genetic variants, such as the APOBEC3A_B deletion polymorphism, has been correlated with an increased frequency of somatic mutations and higher cancer risk, with variable implications depending on population characteristics [7,8,9].

While APOBEC activity is often protective in the context of viral infections, it can also be manipulated by viruses, such as HPV, to facilitate genetic diversification and adaptation. Simultaneously, the genomic alterations promoted by these enzymes in host cells have implications not only for the initiation but also for the progression of cancer [10] since elevated APOBEC3 activity driven by HPV oncogene expression is likely a significant contributor to somatic mutagenesis in HPV-positive cancers [11].

This review aims to explore the molecular mechanisms and biological implications of APOBEC proteins, highlighting their dual functions as mediators of antiviral defense and potential agents of tumorigenic mutagenesis. The clinical implications of genetic variants in these enzymes, particularly in HPV-associated cancers, are discussed, alongside their potential applications in prediction, diagnosis, and therapy.

## 2. APOBEC Superfamily

The human APOBEC protein family comprises 11 members, with respective genes located at different chromosomes as follows: activation-induced cytidine deaminase (AID) and APOBEC1 (chromosome 12), APOBEC2 (chromosome 6), 7 APOBEC3 proteins (i.e., A3A, A3B, A3C, A3DE, A3F, A3G, and A3H) (chromosome 22), and APOBEC4 (chromosome 1) [12,13,14,15,16,17].

**Figure 1:**
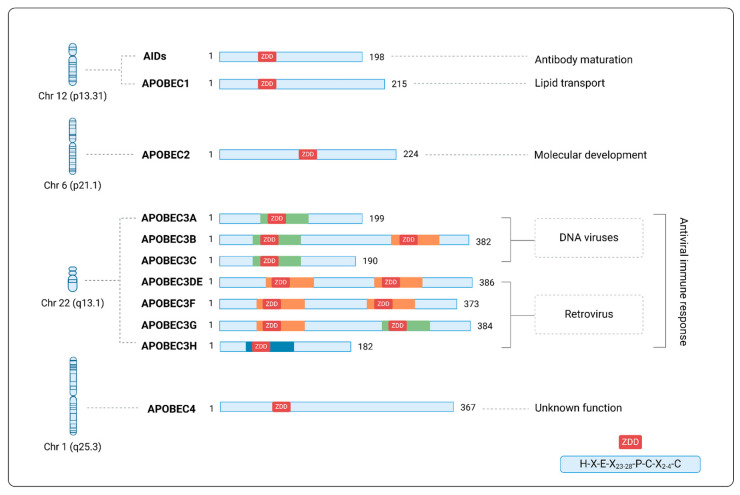
Structure and functional characteristics of APOBECs. Schematic representation of the structure of the APOBEC family enzymes, containing catalytic domains formed by zinc-dependent deaminases (ZDDs). Green, orange, and blue markings represent variations in the canonical ZDD structure (histidine-X-glutamic acid-X23-28-proline-cysteine-X2-4-cysteine, where X represents any amino acid), shown in the right column of the figure. The chromosomal location of each gene is indicated on the left.

The APOBEC genes encode structurally similar enzymes, which contain catalytic domains (CDs) formed by zinc-dependent deaminases (ZDDs), characterized by conserved amino acid sequences. These enzymes have tissue-specific functions, playing roles in immune responses, metabolism, and gene expression regulation through cytidine deamination activity [15,18] (Figure 1).

**Figure 2:**
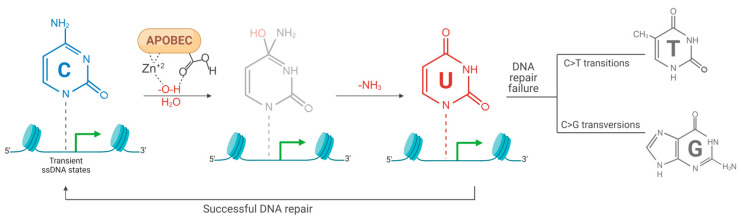
Deamination of cytidines. Chemical representation of the deamination process, which involves the hydrolysis of the amino group (NH_2_) at position 4 of deoxycytidine, resulting in the formation of deoxyuridine (C>U), catalyzed by the APOBEC enzyme. The uridine formed after deamination, with efficient repair, can return to its original state (U>C). When DNA repair fails, abasic sites are generated, promoting transitions (C>T) and transversions (C>G) that are perpetuated in mutations in the following replication cycle.

Cytidine deamination occurs when an APOBEC enzyme catalyzes the hydrolysis of the amino group at position 4 of cytidine, converting it into uridine (C>U) within specific consensus sequences in single-stranded DNA (ssDNA) or RNA [16,19] (Figure 2).

AID is expressed in activated B cells within germinal centers, where it catalyzes recombination and somatic hypermutation in the genomic DNA of immunoglobulin variable regions. This facilitates class switching and antibody diversification, which are essential for adaptive immunity [20].

APOBEC1 is predominantly expressed in the enterocytes of the gastrointestinal tract and is responsible for the post-transcriptional editing of apolipoprotein B (APOB) mRNA. APOBEC1 introduces a premature stop codon (CAA>UAA) through C>U modification, leading to early translation termination and the formation of a truncated ApoB protein, known as ApoB48, which contains 48% of the original sequence. This protein is involved in dietary lipid transport and forms part of the structure of chylomicrons [21,22]. The antiviral activity of APOBEC1 is being investigated [1].

APOBEC2 is related to proper muscle differentiation and development and is primarily expressed in the heart and skeletal muscles; however, its specific function remains unclear [17,23]. APOBEC4 is predominantly expressed in the testes, and its role remains unclear; however, recent studies suggest potential involvement in ribosome biogenesis [1,20,24].

Finally, APOBEC3 is recognized as a key component of the innate immune response to viruses, restricting viral replication and dissemination [25]. Its role in HIV infection, as well as infections caused by other viruses such as HPV, will be further explored in subsequent sections, offering a comprehensive understanding of its dual role in immune response and cellular processes. Building on this understanding, Jang and colleagues [26] investigated the cellular interactions and potential roles of APOBEC3 enzymes. They identified RNA-mediated interactions involving APOBEC3C, APOBEC3H (haplotypes I and II), and APOBEC3G in a distinct manner with specific spliceosome proteins, as well as interactions between APOBEC3G and APOBEC3H (haplotype I) with proteins associated with tRNA methylation and ncRNA export from the nucleus [26].

A3B has been implicated in restricting Hepatitis B Virus (HBV) replication in hepatocytes, and high expression has been seen in the lungs and spleen. A3B may also be associated with malaria incidence in endemic regions of Africa [27,28]. Additionally, RNA-independent protein–protein interactions have been observed, particularly between APOBEC3B and prefoldin 5 (PFD5), which inhibit the PFD5-mediated-degradation of the oncogene cMyc, suggesting a role for APOBEC3B in cancer pathways [26].

## 3. Antiviral Activity of APOBEC3 Proteins

APOBEC3 proteins are part of the first line of defense against many viruses. The expression of these proteins is triggered by viral recognition, primarily through endosomal Toll-like receptors (TLRs), which stimulate the production of pro-inflammatory cytokines and type I interferons, subsequently activating the expression of APOBEC3 genes [23].

The interaction of these defense proteins with viral families is heterogeneous and complex owing to the structural and mechanistic diversity of these pathogens [29]. Additionally, the intracellular localization of A3 proteins determines their restriction targets. A3A, A3C, and A3H are nucleocytoplasmic, while A3B is predominantly nuclear, and A3DE, A3F, and A3G are cytoplasmic [17]. Consequently, A3G, A3DE, A3F, and A3H are closely associated with retroviral restriction, whereas A3A, A3B, and A3C restrict DNA viruses, such as HPV [30,31].

APOBEC3 activity against HIV is the most well established in the literature, with A3G being the primary protein involved in HIV infection and the first reported to neutralize HIV in T cells [25,32]. During the virus’s replication cycle, complementary DNA (cDNA) is synthesized from viral genomic RNA through reverse transcription. During this process, A3G deaminates cytosines in the cDNA, converting them into uracils, which, in turn, leads to the incorporation of adenines into the viral DNA, resulting in G>A mutations in the HIV genome. The accumulation of these mutations causes genetic information loss, leading to defective or degraded virions due to the formation of premature stop codons [23,25,33].

An alternative outcome of the interaction between A3G and HIV is immune evasion through the viral infectivity factor (Vif), which induces the degradation of A3G via the ubiquitin–proteasome pathway. When A3G neutralization is incomplete, this enzyme, along with less mutagenic A3 variants (A3DE, A3F, and A3H), serves as a source of nonsynonymous mutations in the viral genome, promoting HIV diversification, evolution, and immune escape [29,34].

In addition to playing a dominant role in anti-HIV activity, A3G, together with A3F, A3C, and A3B, is proposed to help prevent simian immunodeficiency virus (SIV) infection in humans [35]. Additionally, A3A is a potent inhibitor of adeno-associated virus type 2, with its activity linked to specific DNA-binding loops. Interferon-alpha also enhances the expression of two A3A isoforms in monocytes and macrophages, suggesting that A3A may protect these cells from viral and retroelement DNA [36].

Hypotheses regarding A3 activity, specifically, C-to-U edits, have been developed from genomic variation analyses of acute respiratory syndrome coronavirus 2 (SARS-CoV-2). There is no evidence of A3 enzymes inhibiting the viral cycle; however, APOBEC3A, APOBEC1, and APOBEC3G may deaminate specific sites in SARS-CoV-2 RNA [37].

APOBEC proteins can hyperedit HBV DNA and inhibit viral replication. While A3 proteins are normally present at low levels in the liver, their expression is strongly induced by interferon-alpha and interferon-gamma, generated in chronically active infections. These A3 deaminases are incorporated into nascent HBV capsids, where they modify newly synthesized DNA by deaminating cytidine into uracil after the reverse transcription of pregenomic RNA. This modified HBV DNA is either degraded or incorporates G-to-A mutations into the viral genome, disrupting coding sequences. Even A3 proteins with lost cytidine deaminase activity can still inhibit HBV replication, indicating alternative mechanisms of inhibition [38]. HBV integrates into the host DNA and increases the risk of hepatocellular carcinoma (HCC) by promoting genomic instability. In this context, A3 enzymes exhibit inconsistent patterns of expression alteration, with A3B being upregulated in tumor hepatic tissues and showing more significant evidence [39].

Viral infections, such as those caused by the Epstein–Barr Virus (EBV) and Kaposi sarcoma-associated herpesvirus (KSHV), are associated with the tumorigenesis of Burkitt lymphoma, nasopharyngeal cancer, and Kaposi sarcoma [40]. EBV and KSHV are dsDNA viruses with low susceptibility to deamination by A3 enzymes. Additionally, they possess mechanisms to inhibit A3A and A3B through ribonucleotide reductases, which promote the formation of cytoplasmic aggregates of both A3 enzymes [41].

A3A and A3B are frequently mutated in virus-associated tumor tissues, and the presence of chronic viral infection can lead to increased expression and altered activity in A3, resulting in DNA deamination in the host [42,43]. The hypermutation of mitochondrial DNA promoted by A3B, which is upregulated in nasopharyngeal cells, is being investigated as a key factor in metastases to the neck region in patients with nasopharyngeal cancer [44].

The editing activity of A3 has also been reported in HPV, where both DNA strands of this virus, in transient single-stranded states during transcription or gene replication, are susceptible to editing by A3A, A3B, A3C, and A3H [23,45]. In vitro studies have demonstrated hypermutation by A3A in the long control region (LCR) and the E1 and E2 genes of HPV during the initial phase of infection [45,46]; however, this has not been correlated with antiviral activity, likely because HPV exists in an episomal form during its productive phase, limiting APOBEC’s access to its ssDNA [47]. Another study suggests that A3A directly inhibits HPV infectivity in human cervical keratinocytes but does not specify the restriction sites or viral cycle stages where this activity is most effective [30].

Alternative mechanisms by which APOBEC antiviral activity may target HPV that have been proposed include the C>U editing of viral transcripts encoding the L1 and L2 capsid proteins by A3A, reducing HPV infectivity [19]. The current literature does not clarify whether HPV neutralizes or manipulates A3 enzyme activity to favor its infectivity [48]. On the one hand, cervical infections with HPV genomes bearing A3 signature mutations are more likely to be eliminated [48]. On the other hand, the high-risk HPV (HR-HPV) E6 protein can upregulate A3B gene expression and A3B activity in keratinocytes, which is necessary to sustain elevated A3B levels in infected cancer cells [49].

It is hypothesized that high-risk HPV types did not evolve to evade A3 responses, potentially utilizing them to generate viral genetic diversity [49,50]. This is evidenced by the fact that while many A3-induced mutations are deleterious to the virus, such as stop codon formation, others are neutral or increase viral fitness through amino acid substitution. Moreover, in cervical keratinocytes, HR-HPV underwent slow, random selection due to A3 restriction, significantly depleting A3 target sequences (TCs) in the virus [51].

Unlike HIV, HPV does not appear to possess a specific protein that is able to inhibit A3 activity but has instead developed strategies to establish persistent viral infections. Viral clones selected in this context have reduced A3 restriction sites, both through target sequence depletion and integration into the host genome, reducing free ssDNA. The lack of A3 access to HPV may contribute to off-target somatic mutations in the host genome [13,50].

## 4. Hypermutation in the Cellular Genome

The hypermutation caused by cytidine deamination via A3 enzymes, which plays a role in the antiviral immune response, is also implicated in cellular genome alterations that promote tumor progression [52]. Various studies report the association between the overexpression of A3 enzymes and several human cancers, including bladder, cervical, breast, head and neck, and lung cancers [53,54,55,56,57,58]. Key evidence supporting this hypothesis includes the frequent detection of clustered, strand-coordinated A3 signature mutations (*kataegis*) in cancerous tissues, frequent nuclear DNA damage caused by A3A and A3B, and the correlation between the mRNA expression of these enzymes and the mutational burden in cancer cells [53]. Further evidence for the relationship of A3A with cancer is the loss of mutational signature accumulation following the deletion of A3A and, to a lesser extent, A3B in cancer cell lines [59].

Petljak et al. investigated the role of endogenous APOBEC3 cytidine deaminases in generating prevalent mutational signatures in human cancer genomes by deleting *APOBEC3A* and *APOBEC3B* genes in cancer cell lines naturally exhibiting APOBEC3-associated mutational signatures, demonstrating that *APOBEC3A* deletion significantly reduced these signatures, while the dual deletion of *APOBEC3A* and *APOBEC3B* further decreased mutation burdens without completely eliminating them. Intriguingly, *APOBEC3B* deletion increased *APOBEC3A* protein levels, activity, and mutagenesis in certain cell lines, suggesting a regulatory role for *APOBEC3B* in restraining *APOBEC3A*-dependent mutagenesis. These findings support the hypothesis that *APOBEC3A* is the primary contributor of mutational signatures in cancer [59].

Cytidine deamination in the cellular genome predominantly occurs during transient single-stranded DNA (ssDNA) states, which arise during replication, recombination, and gene transcription [12]. A3A and A3B access the lagging strand of DNA, preferring the 5′-TC motif (with C as the mutated cytidine), inducing cytidine-to-uridine transitions [19]. This modification can lead to genomic DNA breaks, causing genotoxicity and triggering DNA repair signaling pathways. These modifications are removed by base excision repair (BER). BER typically removes incorrect uracil bases from DNA. If this repair process is unsuccessful before the next round of DNA replication, the uracil can be mistakenly incorporated into the new DNA strand, leading to the establishment of mutations. This failure results in C>T transitions, C>G transversions, or double-strand breaks [14].

A large proportion of dispersed and clustered C>T mutations across various types of cancer have been attributed to A3B [57,60], particularly those affecting the phosphatidylinositol-4,5-bisphosphate 3-kinase catalytic subunit alpha gene (*PIK3CA*) [19]. However, A3A is a more potent DNA deaminase than A3B, and the derivation of separate A3A and A3B mutational signatures—in which A3A has been demonstrated to preferentially target YTCN sites (where Y = pyrimidine), whereas A3B preferentially targets RTCN sites (where R = purine)—suggest A3A accounts for a significantly higher proportion of the somatic mutations seen in HPV-associated cancers than A3B [61,62]. Indeed, both helical domain hotspot mutations in *PIK3CA* (E542 and E545) that are thought to be caused by APOBEC3 activity lie within the YTCA context preferred by A3A [63].

The intranuclear concentration of A3A is directly proportional to its avidity for DNA binding. At higher concentrations, A3A tends to form homodimers, which have a higher affinity for ssDNA, while at lower concentrations, A3A remains monomeric, reducing its association with DNA [64]. A3A is considered a potent mutagenic agent, especially when one considers that its constitutive expression is significantly higher in the mucosal epithelium compared to the cutaneous epithelium [51].

## 5. APOBEC3A_B Polymorphism

**Figure 3:**
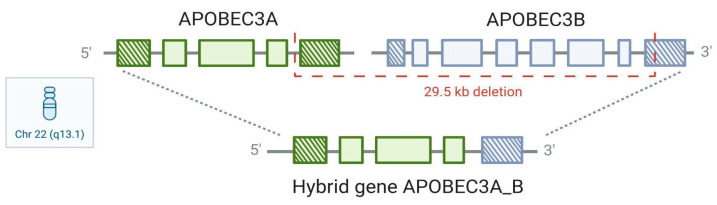
Structure of the APOBEC3A_B deletion polymorphism. APOBEC3A (A3A) and APOBEC3B (A3B) locus together with allelic variation following the 29.5 kb deletion spanning the fifth exon of A3A and the eighth exon of A3B on chromosome 22. Representation of the hybrid A3A_B gene generated by the deletion.

In 2007, Kidd et al. described a deletion polymorphism (*APOBEC3A_B*, hereafter referred to as A3A_B) that displays remarkable stratification across the global population—rising from a frequency of less than 1% in African populations and 6% in Europeans to 37% and 58% in East Asians and Amerindians, respectively, and near fixation in Oceania [28]. This polymorphism likely originated from an ancient non-allelic homologous recombination event on chromosome 22, resulting in the deletion of 29.936 kb between the fifth exon of A3A and the eighth exon of A3B. The deletion breakpoints lie within a 350 bp sequence in two highly identical regions flanking the deletion [65]. The product of this variation is a hybrid A3A_B gene, where the coding sequence and the regulatory elements are identical to A3A, but the untranslated 3′ region (3′UTR) is derived from the A3B gene (Figure 3). Thus, A3B is deleted, and the resulting protein from the hybrid A3A_B allele is identical to that of wild-type A3A but with altered post-transcriptional regulation [27,29]. Despite evidence implicating A3B as a source of APOBEC mutational signatures in breast cancer [60], Nik-Zainal et al. (2014) demonstrated that breast cancers from patients with at least one copy of the A3A_B allele had significantly more APOBEC-associated mutations than those from patients homozygous for the wild-type allele (A3B) [9]. These tumors were also more likely to be ‘hypermutated’ (statistical outliers harbouring a significantly increased total mutation burden compared with the overall cohort.).

One possible explanation for the apparent increase in APOBEC-mediated mutagenesis in tumor cells lacking A3B is the differential regulation of A3A expression in the context of the A3A_B hybrid allele. Indeed, the A3A_B hybrid transcript has been reported to be 10 to 20 times more stable and expressed at higher levels than the wild-type transcript when expressed from a heterologous promoter in transfection experiments [66], and increased A3A expression was observed in oral squamous cell carcinomas (OSCC) in a Taiwanese population with A3B homozygous deletion compared to individuals with heterozygous deletion and individuals with A3B present [58]. Gene expression is post-transcriptionally regulated by specific microRNAs (miRNAs) that target 3′UTRs to induce mRNA repression or degradation, and a study evaluating 30 miRNAs potentially regulating A3A and A3B via in silico analysis concluded that only 8 miRNAs specific to A3B can regulate the A3A_B transcript [67]. In addition to the loss of miRNA binding sites that would otherwise mediate A3A repression, there is evidence that miRNAs specific to the A3B 3′UTR are downregulated in tumors from A3A_B carriers. Examples include the loss of miR-34b-3p in cervical cancer and miR-409 in OSCC [58,67].

Alternatively, haplotype I of the highly polymorphic A3H gene, which encodes a predominantly nuclear-localized isoform, has been proposed as a source of the APOBEC signature mutations observed in A3A_B carriers [54]. Although A3H is not genetically linked to A3A or A3B, the frequency of A3H-I is inversely correlated with that of A3B, suggesting a possible functional redundancy. Among seventeen breast tumors sequenced by the Cancer Genome Atlas (TCGA) project, which were shown by Nik-Zainal and colleagues [9] to be homozygous for A3A_B, only three cases were also carriers of A3H-I. Notably, these cases displayed the highest burden of APOBEC signature mutations [54]. While sequencing much larger cohorts containing more A3A_B carriers is required to confirm the potential involvement of A3H in cancer, it is noteworthy that the deletion of A3A and A3B from breast cancer cell lines greatly reduces but did not entirely eliminate the accrual of APOBEC signature mutations [59]. Together, these findings clearly suggest that further studies are required to understand the contribution of A3H and/or other members of the family to somatic mutagenesis in cancer [54].

## 6. APOBEC3A_B Deletion Polymorphism Frequency, Population Variation, and Cancer Risk

The A3A_B deletion polymorphism is linked to C>T and C>G mutations. Initially identified in breast cancer, these signature mutations were later found in various other cancer types, indicating that A3 activity extends beyond breast tissue [9]. Multiple studies have associated the A3A_B deletion with cancers, including breast [68], bladder, liver [9], head and neck, lung [69], cervical [67], and ovarian [70].

An association between A3A_B deletion and increased breast cancer risk was shown in a Chinese population [71]; the same association was shown in small studies on Iranian [72], Malaysian [73], and European American [74] populations. However, this association was not seen in studies performed on Swedish [75], Indian [67], and Moroccan [76] populations. Furthermore, a study on a South Indian population showed no association between breast, cervical, or oral cancer and A3A_B deletion. Interestingly, the same study indicated a higher incidence of this deletion polymorphism in women than in men, both in oral cancer samples and controls; however, it is unclear as to why that might be the case [67].

Meta-analyses, including these and other studies, suggest that the association of A3A_B with increased cancer susceptibility is dependent on the ethnic characteristics of the population studied, as might be expected based on the high variability in the incidence of this polymorphism reported by Kidd et al. [27,65,67,77]. These association studies indicate conflicting results concerning the effects of A3A_B deletion on Caucasian populations, mirrored by the frequency distribution of the deletion.

The association between A3A_B and malaria susceptibility in humans has been investigated in endemic regions [27,28]. In a study on Indian populations, the deletion allele was associated with a higher probability of infection by Plasmodium falciparum, and a low deletion frequency was seen in individuals living in endemic regions of India [28]. Thus, it seems that A3A_B is under selective pressure and that its worldwide distribution mirrors the spread of certain pathogens such as Plasmodium falciparum [28]. This might provide an explanation for the frequency of the A3A_B deletion worldwide: as populations moved out of Africa and migrated toward Europe and then Asia, the protective action of A3B against the pathogen was no longer needed. Additionally, it has been speculated that HIV infection might also play a role in the distribution of the deletion, together with malaria [28]. However, the increase in A3A_B frequency to the point of approaching fixation in Oceania may also suggest positive selection for the allele, and population movement might not have been the only influence on A3A_B polymorphism; other A3 genes also show intricate polymorphism, and it has recently been hypothesized that they might also be involved in increased cancer risk [65].

Allelic variations in A3 genes may increase mutagenesis, change HPV infection persistence, and contribute to cellular immortalization. These variations are potential markers for cancer susceptibility and prognosis [50,78].

## 7. HPV-Related Cancers and APOBEC3A_B Polymorphism

APOBEC3 activity is the predominant source of somatic mutations in cervical cancer [60,79,80,81,82] in HPV-associated head and neck squamous cell carcinoma (HNSCC) [63,83] and in HPV-associated penile cancer [84]. The increased expression of A3B appears to be a determining factor of mutations in bladder cancer, breast cancer, lung squamous cell carcinoma, and lung adenocarcinoma, as well as head and neck and cervical cancers [60].

A3B is widely reported to be a protein that promotes cell proliferation and migration while inhibiting cancer cell apoptosis through the p53-mediated signaling pathway [85]. The first study to establish a mechanistic link between HPV infection and the upregulation of DNA cytidine deaminase A3B demonstrated that the E6 oncoprotein is essential for maintaining elevated A3B levels in HPV-positive cancer cell lines. In this context, high-risk E6 inactivates TP53, leading to the de-repression of A3B gene transcription [86].

It is worth noting that there are ongoing discussions regarding the actual contribution of A3B to cancer. Recently, an analysis investigating the mutational signatures of A3A and A3B in the cancer genome demonstrated that the signatures were characteristic of A3A rather than A3B, countering previous studies [87].

In HPV-associated neoplasms, the inflammatory microenvironment likely contributes to elevated A3A expression via type 1 interferon signaling, facilitating the accumulation of driver somatic mutations [13].

**Figure 4:**
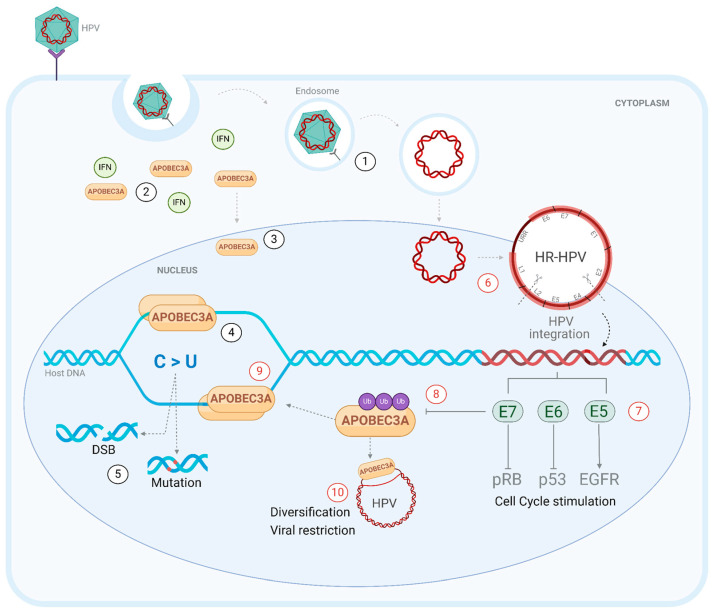
APOBEC3A-mediated mutagenesis. Schematic representation of the interaction of APOBEC3A (A3A) with the cellular genome on the left (black numbers) and with human papillomavirus (HPV) DNA on the right (red numbers). The recognition of HPV in endosomal vesicles (1) induces the transcription of interferons that stimulate the expression of A3A genes (2). Cytoplasmic A3A can access the nucleus (3) and interact with cellular DNA through the formation of homodimers (4). A3A deaminates target cytidines (5′TC) in the genome, which may or may not be repaired, resulting in double-strand breaks (DSBs) or the accumulation of somatic mutations (5) that promote cellular alterations. The nuclear access of high-risk HPV (HR-HPV) (6) can lead to integration into cellular DNA, causing the de-repression of oncoproteins E7, E6, and E5, which act on targets such as pRb, p53, and EGFR, promoting cell cycle stimulation (7). E7 can associate with A3A and prevent its degradation (8), enhancing the interaction of this protein with viral DNA during transient single-stranded states (9). The deamination of HPV by A3A aims to eliminate the virus through restriction but can also lead to genome evolution and viral diversity when mutations caused by A3A are non-deleterious (10).

Increased nucleocytoplasmic levels of A3A could increase the formation of homodimers that interact with cellular genomic ssDNA and are correlated with increased C>T and C>G signature mutations in TC dinucleotides in cancer cells [88] (Figure 4). DNA deamination by A3A can result in missense somatic mutations, altering the sequence of the amino acids to be encoded [89]. Some of these mutations will result in the generation of neoantigens—variant peptides present on the surface of tumor cells in class 1 MHC (major histocompatibility complex) and recognized as foreign by the immune system [88]. Thus, A3A may enhance the immune response against cancer cells and correlate with the response to cancer therapy [89].

HNSCC has HPV as one of its main risk factors, independently associated with the tumorigenesis of oropharyngeal epithelial cells [16]. Mutational profile analyses of HPV-positive HNSCC have identified APOBEC signature mutations, including TC>TT and TC>TG [63], as well as a positive correlation between A3A expression and HPV integration into the host cell DNA [30]. The presence of HPV in HNSCC, by modulating A3A activity, promotes the accumulation of mutations in cancer driver genes, such as the PIK3CA gene, conferring a proliferative advantage to these cells [63]. As reviewed by Dananberg and colleagues [3], who summarized the key evidence linking APOBEC mutagenesis to carcinogenesis across different tissues, APOBEC activity may be a significant contributor to the genomic instability associated with cancer [3].

In cervical cancer, replication stress exacerbates A3-induced mutations, and the expression of these enzymes is upregulated in cancerous cells [90,91]. The disruption of the G1/S cell cycle checkpoint caused by the combined action of E6 and E7 from high-risk HPV (HR-HPV) generates replication stress, increasing the exposure of cellular ssDNA at stalled replication forks and potentially rendering it susceptible to A3 restriction [16,19]. HPV oncoproteins can upregulate A3B and A3A: E6 inactivates p53, leading to the de-repression of A3B [86,92,93], and E7 prevents the proteasomal degradation of A3A through a protein stabilization mechanism in immortalized keratinocytes [10] (Figure 4). Replication stress may also lead to increased A3A gene expression via NFkB signaling, as demonstrated in the HPV-negative HNSCC cell line, BICR6 [94]. In the context of HPV infection, the E7 oncoprotein stabilizes the A3A protein encoded by A3A and/or A3A_B alleles, amplifying its hypermutator potential in the cellular genome [10]. Furthermore, the immune system response to viral infection promotes an interferon-rich environment that stimulates the transcription of A3A [95]. Finally, a recent study that combined single-cell and spatial transcriptomics analysis with the immunohistochemistry of HPV+ HNSCC tissues demonstrated A3A and A3B expression in distinct compartments; A3A expression was largely confined to more differentiated tumor cells, whereas A3B was restricted to proliferating tumor cells entering mitosis [96]. It remains to be seen whether A3A expression extends to both compartments in the context of the A3A_B allele, but if so, increased A3A levels in cells undergoing DNA replication might well be expected to result in increased mutagenesis.

Regarding cervical cancer, this polymorphism may potentially be protective, by increasing viral restriction and immune system activation, as demonstrated in an in vitro study where A3A inhibited the proliferation, migration, and invasion of cervical cancer cells [97]. Paradoxically, A3A_B deletion may also drive viral diversity depending on the virus type, A3A intracellular levels, and accumulation of somatic mutations, which predispose to cervical neoplastic changes [70]. Furthermore, HR-HPV types enhance A3A expression in the cervical epithelium [10] (Figure 4).

Breast cancers in patients with the A3A_B deletion display an ‘immune hot’ tumor microenvironment—characterized by increased T-lymphocyte infiltration possibly more responsive to immunotherapy [98]—and the enrichment of APOBEC3 signature mutations has similarly been linked to an inflamed microenvironment in HPV-associated HNSCC [99]. Further research is needed to confirm whether A3A_B and/or increased APOBEC3 activity might predict responses to immunotherapy in HPV-associated cancer. It was recently shown that A3A might be an important prognostic marker, albeit only when the A3B deletion polymorphism is present. This was seen in a small Taiwanese population with oral cancer; nonetheless, this study can prompt further genomic studies on different cancers [58]. A potential treatment with anti-PD1 is also suggested in lung cancers rich in APOBEC mutation signatures [100], providing further evidence of the need to investigate APOBEC in future therapies.

## 8. Conclusions

In summary, like A3A and A3B, the A3A_B hybrid gene is a likely source of somatic mutations contributing to tumorigenesis in cancer. A3A and A3B are important antiviral proteins; however, high cellular expression can be dangerous, as it can cause mutations in the genome, leading to cancer formation. Currently, A3A and A3B mutational signatures are implicated in specific cancers; however, further studies are needed in order to make clear links and correlations. In addition, the peculiar A3B deletion phenotype adds complexity to the picture: a deletion of a highly mutagenic gene predicts a reduction in mutations and cancer incidence; however, A3B deletion and consequent A3A_B hybrid gene formation increase cancer risk only in specific populations.

Further studies are, hence, needed to understand A3A_B gene functions within the cell and how this might affect the cell cycle and proliferation, as well as how this phenotype might affect viral infection.

## Data Availability

This study is a literature review and does not involve the generation or analysis of new datasets. All data and materials discussed in this manuscript are based on previously published studies, which are cited throughout the text.

## References

[1] Mertz T.M., Collins C.D., Dennis M., Coxon M., Roberts S.A. (2022). APOBEC-Induced Mutagenesis in Cancer. Annu. Rev. Genet..

[2] Pecori R., Di Giorgio S., Paulo Lorenzo J., Nina Papavasiliou F. (2022). Functions and Consequences of AID/APOBEC-Mediated DNA and RNA Deamination. Nat. Rev. Genet..

[3] Dananberg A., Striepen J., Rozowsky J.S., Petljak M. (2024). APOBEC Mutagenesis in Cancer Development and Susceptibility. Cancers.

[4] Smith N.J., Fenton T.R. (2019). The APOBEC3 Genes and Their Role in Cancer: Insights from Human Papillomavirus. J. Mol. Endocrinol..

[5] Hata A.N., Larijani M. (2024). Targeting APOBECs in Cancer: It’s about Timing. Cancer Cell.

[6] Landry S., Narvaiza I., Linfesty D.C., Weitzman M.D. (2011). APOBEC3A Can Activate the DNA Damage Response and Cause Cell-Cycle Arrest. EMBO Rep..

[7] Chervova A., Fatykhov B., Koblov A., Shvarov E., Preobrazhenskaya J., Vinogradov D., Ponomarev G.V., Gelfand M.S., Kazanov M.D. (2021). Analysis of Gene Expression and Mutation Data Points on Contribution of Transcription to the Mutagenesis by APOBEC Enzymes. NAR Cancer.

[8] Castilha E.P., Curti R.R.d.J., de Oliveira J.N., Vitiello G.A.F., Guembarovski R.L., Couto-Filho J.d., Oliveira K.B.d. (2023). APOBEC3A/B Polymorphism Is Not Associated with Human Papillomavirus Infection and Cervical Carcinogenesis. Pathogens.

[9] Nik-Zainal S., Wedge D.C., Alexandrov L.B., Petljak M., Butler A.P., Bolli N., Davies H.R., Knappskog S., Martin S., Papaemmanuil E. (2014). Association of a Germline Copy Number Polymorphism of APOBEC3A and APOBEC3B with Burden of Putative APOBEC-Dependent Mutations in Breast Cancer. Nat. Genet..

[10] Westrich J.A., Warren C.J., Klausner M.J., Guo K., Liu C.-W., Santiago M.L., Pyeon D. (2018). Human Papillomavirus 16 E7 Stabilizes APOBEC3A Protein by Inhibiting Cullin 2-Dependent Protein Degradation. J. Virol..

[11] Warren C.J., Santiago M.L., Pyeon D. (2022). APOBEC3: Friend or Foe in Human Papillomavirus Infection and Oncogenesis?. Annu. Rev. Virol..

[12] Green A.M., Weitzman M.D. (2019). The Spectrum of APOBEC3 Activity: From Anti-Viral Agents to Anti-Cancer Opportunities. DNA Repair.

[13] Chen L., Qiu X., Zhang N., Wang Y., Wang M., Li D., Wang L., Du Y. (2017). APOBEC-Mediated Genomic Alterations Link Immunity and Viral Infection during Human Papillomavirus-Driven Cervical Carcinogenesis. Biosci. Trends.

[14] Henderson S., Fenton T. (2015). APOBEC3 Genes: Retroviral Restriction Factors to Cancer Drivers. Trends Mol. Med..

[15] Revathidevi S., Murugan A.K., Nakaoka H., Inoue I., Munirajan A.K. (2021). APOBEC: A Molecular Driver in Cervical Cancer Pathogenesis. Cancer Lett..

[16] Riva G., Albano C., Gugliesi F., Pasquero S., Pacheco S.F.C., Pecorari G., Landolfo S., Biolatti M., Dell’Oste V. (2021). HPV Meets APOBEC: New Players in Head and Neck Cancer. Int. J. Mol. Sci..

[17] Salter J.D., Bennett R.P., Smith H.C. (2016). The APOBEC Protein Family: United by Structure, Divergent in Function. Trends in Biochemical Sciences.

[18] Cervantes-Gracia K., Gramalla-Schmitz A., Weischedel J., Chahwan R. (2021). APOBECs Orchestrate Genomic and Epigenomic Editing across Health and Disease. Trends Genet..

[19] Warren C., Westrich J., Doorslaer K., Pyeon D. (2017). Roles of APOBEC3A and APOBEC3B in Human Papillomavirus Infection and Disease Progression. Viruses.

[20] Rebhandl S., Huemer M., Greil R., Geisberger R. (2015). AID/APOBEC Deaminases and Cancer. Oncoscience.

[21] Lo C.C., Coschigano K.T. (2020). ApoB48 as an Efficient Regulator of Intestinal Lipid Transport. Front. Physiol..

[22] Nakajima K., Nagamine T., Fujita M.Q., Ai M., Tanaka A., Schaefer E. (2014). Apolipoprotein B-48: A Unique Marker of Chylomicron Metabolism. Advances in Clinical Chemistry.

[23] Vieira V.C., Soares M.A. (2013). The Role of Cytidine Deaminases on Innate Immune Responses against Human Viral Infections. BioMed Res. Int..

[24] McCool M.A., Bryant C.J., Abriola L., Surovtseva Y.V., Baserga S.J. (2024). The Cytidine Deaminase APOBEC3A Regulates Nucleolar Function to Promote Cell Growth and Ribosome Biogenesis. PLoS Biol..

[25] Uriu K., Kosugi Y., Ito J., Sato K. (2021). The Battle between Retroviruses and APOBEC3 Genes: Its Past and Present. Viruses.

[26] Jang G.M., Sudarsan A.K.A., Shayeganmehr A., Munhoz E.P., Lao R., Gaba A., Rodríguez M.G., Love R.P., Polacco B.J., Zhou Y. (2024). Protein Interaction Map of APOBEC3 Enzyme Family Reveals Deamination-Independent Role in Cellular Function. Mol. Cell. Proteom..

[27] Kidd J.M., Newman T.L., Tuzun E., Kaul R., Eichler E.E. (2007). Population Stratification of a Common APOBEC Gene Deletion Polymorphism. PLoS Genet..

[28] Jha P., Sinha S., Kanchan K., Qidwai T., Narang A., Singh P.K., Pati S.S., Mohanty S., Mishra S.K., Sharma S.K. (2012). Deletion of the APOBEC3B Gene Strongly Impacts Susceptibility to Falciparum Malaria. Infect. Genet. Evol..

[29] Sadeghpour S., Khodaee S., Rahnama M., Rahimi H., Ebrahimi D. (2021). Human APOBEC3 Variations and Viral Infection. Viruses.

[30] Warren C.J., Xu T., Guo K., Griffin L.M., Westrich J.A., Lee D., Lambert P.F., Santiago M.L., Pyeon D. (2015). APOBEC3A Functions as a Restriction Factor of Human Papillomavirus. J. Virol..

[31] Mangeat B., Turelli P., Caron G., Friedli M., Perrin L., Trono D. (2003). Broad Antiretroviral Defence by Human APOBEC3G through Lethal Editing of Nascent Reverse Transcripts. Nature.

[32] Sheehy A.M., Gaddis N.C., Choi J.D., Malim M.H. (2002). Isolation of a Human Gene That Inhibits HIV-1 Infection and Is Suppressed by the Viral Vif Protein. Nature.

[33] Delviks-Frankenberry K.A., Nikolaitchik O.A., Burdick R.C., Gorelick R.J., Keele B.F., Hu W.-S., Pathak V.K. (2016). Minimal Contribution of APOBEC3-Induced G-to-A Hypermutation to HIV-1 Recombination and Genetic Variation. PLoS Pathog..

[34] Sato K., Takeuchi J.S., Misawa N., Izumi T., Kobayashi T., Kimura Y., Iwami S., Takaori-Kondo A., Hu W.-S., Aihara K. (2014). APOBEC3D and APOBEC3F Potently Promote HIV-1 Diversification and Evolution in Humanized Mouse Model. PLoS Pathog..

[35] Yu Q., Chen D., König R., Mariani R., Unutmaz D., Landau N.R. (2004). APOBEC3B and APOBEC3C Are Potent Inhibitors of Simian Immunodeficiency Virus Replication. J. Biol. Chem..

[36] Thielen B.K., McNevin J.P., McElrath M.J., Hunt B.V.S., Klein K.C., Lingappa J.R. (2010). Innate Immune Signaling Induces High Levels of TC-Specific Deaminase Activity in Primary Monocyte-Derived Cells through Expression of APOBEC3A Isoforms. J. Biol. Chem..

[37] Kim K., Calabrese P., Wang S., Qin C., Rao Y., Feng P., Chen X.S. (2022). The Roles of APOBEC-Mediated RNA Editing in SARS-CoV-2 Mutations, Replication and Fitness. Sci. Rep..

[38] Janahi E.M., McGarvey M.J. (2013). The Inhibition of Hepatitis B Virus by APOBEC Cytidine Deaminases. J. Viral Hepatitis.

[39] Luo X., Huang Y., Chen Y., Tu Z., Hu J., Tavis J.E., Huang A., Hu Y. (2016). Association of Hepatitis B Virus Covalently Closed Circular DNA and Human APOBEC3B in Hepatitis B Virus-Related Hepatocellular Carcinoma. PLoS ONE.

[40] Lehle J., Soleimanpour M., Mokhtari S., Ebrahimi D. (2024). Viral Infection, APOBEC3 Dysregulation, and Cancer. Front. Genet..

[41] Cheng A.Z., Moraes S.N., Shaban N.M., Fanunza E., Bierle C.J., Southern P.J., Bresnahan W.A., Rice S.A., Harris R.S. (2021). APOBECs and Herpesviruses. Viruses.

[42] Alexandrov L.B., Kim J., Haradhvala N.J., Huang M.N., Tian Ng A.W., Wu Y., Boot A., Covington K.R., Gordenin D.A., Bergstrom E.N. (2020). The Repertoire of Mutational Signatures in Human Cancer. Nature.

[43] Zhang Y., Chen X., Cao Y., Yang Z. (2021). Roles of APOBEC3 in Hepatitis B Virus (HBV) Infection and Hepatocarcinogenesis. Bioengineered.

[44] Wakae K., Kondo S., Pham H.T., Wakisaka N., Que L., Li Y., Zheng X., Fukano K., Kitamura K., Watashi K. (2020). EBV-LMP1 Induces APOBEC3s and Mitochondrial DNA Hypermutation in Nasopharyngeal Cancer. Cancer Med..

[45] Vartanian J.-P., Guétard D., Henry M., Wain-Hobson S. (2008). Evidence for Editing of Human Papillomavirus DNA by APOBEC3 in Benign and Precancerous Lesions. Science.

[46] Hirose Y., Yamaguchi-Naka M., Onuki M., Tenjimbayashi Y., Tasaka N., Satoh T., Tanaka K., Iwata T., Sekizawa A., Matsumoto K. (2020). High Levels of Within-Host Variations of Human Papillomavirus 16 E1/E2 Genes in Invasive Cervical Cancer. Front. Microbiol..

[47] Wang Z., Wakae K., Kitamura K., Aoyama S., Liu G., Koura M., Monjurul A.M., Kukimoto I., Muramatsu M. (2014). APOBEC3 Deaminases Induce Hypermutation in Human Papillomavirus 16 DNA upon Beta Interferon Stimulation. J. Virol..

[48] Della Fera A.N., Warburton A., Coursey T.L., Khurana S., McBride A.A. (2021). Persistent Human Papillomavirus Infection. Viruses.

[49] Wallace N.A., Münger K. (2018). The Curious Case of APOBEC3 Activation by Cancer-Associated Human Papillomaviruses. PLoS Pathog..

[50] Zhu B., Xiao Y., Yeager M., Clifford G., Wentzensen N., Cullen M., Boland J.F., Bass S., Steinberg M.K., Raine-Bennett T. (2020). Mutations in the HPV16 Genome Induced by APOBEC3 Are Associated with Viral Clearance. Nat. Commun..

[51] Warren C.J., Van Doorslaer K., Pandey A., Espinosa J.M., Pyeon D. (2015). Role of the Host Restriction Factor APOBEC3 on Papillomavirus Evolution. Virus Evol..

[52] Buisson R., Lawrence M.S., Benes C.H., Zou L. (2017). APOBEC3A and APOBEC3B Activities Render Cancer Cells Susceptible to ATR Inhibition. Cancer Res..

[53] Robertson A.G., Kim J., Al-Ahmadie H., Bellmunt J., Guo G., Cherniack A.D., Hinoue T., Laird P.W., Hoadley K.A., Akbani R. (2017). Comprehensive Molecular Characterization of Muscle-Invasive Bladder Cancer. Cell.

[54] Starrett G.J., Luengas E.M., McCann J.L., Ebrahimi D., Temiz N.A., Love R.P., Feng Y., Adolph M.B., Chelico L., Law E.K. (2016). The DNA Cytosine Deaminase APOBEC3H Haplotype I Likely Contributes to Breast and Lung Cancer Mutagenesis. Nat. Commun..

[55] Kim Y., Sun D.S., Yoon J., Ko Y.H., Won H.S., Kim J.S. (2020). Clinical Implications of APOBEC3A and 3B Expression in Patients with Breast Cancer. PLoS ONE.

[56] Cannataro V.L., Gaffney S.G., Sasaki T., Issaeva N., Grewal N.K.S., Grandis J.R., Yarbrough W.G., Burtness B., Anderson K.S., Townsend J.P. (2019). APOBEC-Induced Mutations and Their Cancer Effect Size in Head and Neck Squamous Cell Carcinoma. Oncogene.

[57] Argyris P.P., Wilkinson P.E., Jarvis M.C., Magliocca K.R., Patel M.R., Vogel R.I., Gopalakrishnan R., Koutlas I.G., Harris R.S. (2021). Endogenous APOBEC3B Overexpression Characterizes HPV-Positive and HPV-Negative Oral Epithelial Dysplasias and Head and Neck Cancers. Mod. Pathol..

[58] Chen T.-W., Lee C.-C., Liu H., Wu C.-S., Pickering C.R., Huang P.-J., Wang J., Chang I.Y.-F., Yeh Y.-M., Chen C.-D. (2017). APOBEC3A Is an Oral Cancer Prognostic Biomarker in Taiwanese Carriers of an APOBEC Deletion Polymorphism. Nat. Commun..

[59] Petljak M., Dananberg A., Chu K., Bergstrom E.N., Striepen J., von Morgen P., Chen Y., Shah H., Sale J.E., Alexandrov L.B. (2022). Mechanisms of APOBEC3 Mutagenesis in Human Cancer Cells. Nature.

[60] Burns M.B., Temiz N.A., Harris R.S. (2013). Evidence for APOBEC3B Mutagenesis in Multiple Human Cancers. Nat. Genet..

[61] Chan K., Roberts S.A., Klimczak L.J., Sterling J.F., Saini N., Malc E.P., Kim J., Kwiatkowski D.J., Fargo D.C., Mieczkowski P.A. (2015). An APOBEC3A Hypermutation Signature Is Distinguishable from the Signature of Background Mutagenesis by APOBEC3B in Human Cancers. Nat. Genet..

[62] Jalili P., Bowen D., Langenbucher A., Park S., Aguirre K., Corcoran R.B., Fleischman A.G., Lawrence M.S., Zou L., Buisson R. (2020). Quantification of Ongoing APOBEC3A Activity in Tumor Cells by Monitoring RNA Editing at Hotspots. Nat. Commun..

[63] Henderson S., Chakravarthy A., Su X., Boshoff C., Fenton T.R. (2014). APOBEC-Mediated Cytosine Deamination Links PIK3CA Helical Domain Mutations to Human Papillomavirus-Driven Tumor Development. Cell Rep..

[64] Bohn M.-F., Shandilya S.M.D., Silvas T.V., Nalivaika E.A., Kouno T., Kelch B.A., Ryder S.P., Kurt-Yilmaz N., Somasundaran M., Schiffer C.A. (2015). The SsDNA Mutator APOBEC3A Is Regulated by Cooperative Dimerization. Structure.

[65] Klonowska K., Kluzniak W., Rusak B., Jakubowska A., Ratajska M., Krawczynska N., Vasilevska D., Czubak K., Wojciechowska M., Cybulski C. (2017). The 30 Kb Deletion in the APOBEC3 Cluster Decreases APOBEC3A and APOBEC3B Expression and Creates a Transcriptionally Active Hybrid Gene but Does Not Associate with Breast Cancer in the European Population. Oncotarget.

[66] Caval V., Suspène R., Shapira M., Vartanian J.-P., Wain-Hobson S. (2014). A Prevalent Cancer Susceptibility APOBEC3A Hybrid Allele Bearing APOBEC3B 3′UTR Enhances Chromosomal DNA Damage. Nat. Commun..

[67] Revathidevi S., Manikandan M., Rao A.K.D.M., Vinothkumar V., Arunkumar G., Rajkumar K.S., Ramani R., Rajaraman R., Ajay C., Munirajan A.K. (2016). Analysis of APOBEC3A/3B Germline Deletion Polymorphism in Breast, Cervical and Oral Cancers from South India and Its Impact on MiRNA Regulation. Tumor Biol..

[68] Vitiello G.A.F., Amarante M.K., Banin-Hirata B.K., Campos C.Z., de Oliveira K.B., Losi-Guembarovski R., Watanabe M.A.E. (2019). Transforming Growth Factor Beta Receptor II (TGFBR2) Promoter Region Polymorphism in Brazilian Breast Cancer Patients: Association with Susceptibility, Clinicopathological Features, and Interaction with TGFB1 Haplotypes. Breast Cancer Res. Treat..

[69] Gansmo L.B., Romundstad P., Hveem K., Vatten L., Nik-Zainal S., Lønning P.E., Knappskog S. (2018). APOBEC3A/B Deletion Polymorphism and Cancer Risk. Carcinogenesis.

[70] Gansmo L.B., Sofiyeva N., Bjørnslett M., Romundstad P., Hveem K., Vatten L., Dørum A., Lønning P.E., Knappskog S. (2021). Impact of the APOBEC3A/B Deletion Polymorphism on Risk of Ovarian Cancer. Sci. Rep..

[71] Long J., Delahanty R.J., Li G., Gao Y.-T., Lu W., Cai Q., Xiang Y.-B., Li C., Ji B.-T., Zheng Y. (2013). A Common Deletion in the APOBEC3 Genes and Breast Cancer Risk. JNCI J. Natl. Cancer Inst..

[72] Rezaei M., Hashemi M., Hashemi S.M., Mashhadi M.A., Taheri M. (2015). APOBEC3 Deletion Is Associated with Breast Cancer Risk in a Sample of Southeast Iranian Population. Int. J. Mol. Cell Med..

[73] Wen W.X., Soo J.S.-S., Kwan P.Y., Hong E., Khang T.F., Mariapun S., Lee C.S.-M., Hasan S.N., Rajadurai P., Yip C.H. (2016). Germline APOBEC3B Deletion Is Associated with Breast Cancer Risk in an Asian Multi-Ethnic Cohort and with Immune Cell Presentation. Breast Cancer Res..

[74] Xuan D., Li G., Cai Q., Deming-Halverson S., Shrubsole M.J., Shu X.-O., Kelley M.C., Zheng W., Long J. (2013). APOBEC3 Deletion Polymorphism Is Associated with Breast Cancer Risk among Women of European Ancestry. Carcinogenesis.

[75] Göhler S., Da Silva Filho M.I., Johansson R., Enquist-Olsson K., Henriksson R., Hemminki K., Lenner P., Försti A. (2016). Impact of Functional Germline Variants and a Deletion Polymorphism in APOBEC3A and APOBEC3B on Breast Cancer Risk and Survival in a Swedish Study Population. J. Cancer Res. Clin. Oncol..

[76] Marouf C., Göhler S., Filho M.I.D.S., Hajji O., Hemminki K., Nadifi S., Försti A. (2016). Analysis of Functional Germline Variants in APOBEC3 and Driver Genes on Breast Cancer Risk in Moroccan Study Population. BMC Cancer.

[77] Hashemi M., Moazeni-Roodi A., Taheri M. (2019). Association of APOBEC3 Deletion with Cancer Risk: A Meta-analysis of 26 225 Cases and 37 201 Controls. Asia Pac. J. Clin. Oncol..

[78] Karki R., Pandya D., Elston R.C., Ferlini C. (2015). Defining “Mutation” and “Polymorphism” in the Era of Personal Genomics. BMC Med. Genom..

[79] Alexandrov L.B., Nik-Zainal S., Wedge D.C., Aparicio S.A.J.R., Behjati S., Biankin A.V., Bignell G.R., Bolli N., Borg A., Børresen-Dale A.-L. (2013). Signatures of Mutational Processes in Human Cancer. Nature.

[80] Roberts S.A., Lawrence M.S., Klimczak L.J., Grimm S.A., Fargo D., Stojanov P., Kiezun A., Kryukov G.V., Carter S.L., Saksena G. (2013). An APOBEC Cytidine Deaminase Mutagenesis Pattern Is Widespread in Human Cancers. Nat. Genet..

[81] Ojesina A.I., Lichtenstein L., Freeman S.S., Pedamallu C.S., Imaz-Rosshandler I., Pugh T.J., Cherniack A.D., Ambrogio L., Cibulskis K., Bertelsen B. (2014). Landscape of Genomic Alterations in Cervical Carcinomas. Nature.

[82] Burk R.D., Chen Z., Saller C., Tarvin K., Carvalho A.L., Scapulatempo-Neto C., Silveira H.C., Fregnani J.H., Creighton C.J., Anderson M.L. (2017). Integrated Genomic and Molecular Characterization of Cervical Cancer. Nature.

[83] The Cancer Genome Atlas Network (2015). Comprehensive Genomic Characterization of Head and Neck Squamous Cell Carcinomas. Nature.

[84] Feber A., Worth D.C., Chakravarthy A., de Winter P., Shah K., Arya M., Saqib M., Nigam R., Malone P.R., Tan W.S. (2016). CSN1 Somatic Mutations in Penile Squamous Cell Carcinoma. Cancer Res..

[85] Wei Z., Gan J., Feng X., Zhang M., Chen Z., Zhao H., Du Y. (2022). APOBEC3B Is Overexpressed in Cervical Cancer and Promotes the Proliferation of Cervical Cancer Cells through Apoptosis, Cell Cycle, and P53 Pathway. Front. Oncol..

[86] Vieira V.C., Leonard B., White E.A., Starrett G.J., Temiz N.A., Lorenz L.D., Lee D., Soares M.A., Lambert P.F., Howley P.M. (2014). Human Papillomavirus E6 Triggers Upregulation of the Antiviral and Cancer Genomic DNA Deaminase APOBEC3B. mBio.

[87] Butt Y., Sakhtemani R., Mohamad-Ramshan R., Lawrence M.S., Bhagwat A.S. (2024). Distinguishing Preferences of Human APOBEC3A and APOBEC3B for Cytosines in Hairpin Loops, and Reflection of These Preferences in APOBEC-Signature Cancer Genome Mutations. Nat. Commun..

[88] Pan J., Zabidi M.M.A., Chong B., Meng M., Ng P., Hasan S.N., Sandey B., Bahnu S., Rajadurai P., Yip C. (2021). Germline APOBEC3B Deletion Increases Somatic Hypermutation in Asian Breast Cancer That Is Associated with Her2 Subtype, PIK3CA Mutations and Immune Activation. Int. J. Cancer.

[89] Chen Z., Wen W., Bao J., Kuhs K.L., Cai Q., Long J., Shu X., Zheng W., Guo X. (2019). Integrative Genomic Analyses of APOBEC-Mutational Signature, Expression and Germline Deletion of APOBEC3 Genes, and Immunogenicity in Multiple Cancer Types. BMC Med. Genom..

[90] Wang C., Bai R., Liu Y., Wang K., Wang Y., Yang J., Cai H., Yang P. (2023). Multi-Region Sequencing Depicts Intratumor Heterogeneity and Clonal Evolution in Cervical Cancer. Med. Oncol..

[91] Niyazi M., Han L., Husaiyin S., Aishanjiang A., Guo M., Muhaimati G., Rozi H., Sun H., Lu J., Ma C. (2023). Analysis of Somatic Mutations and Key Driving Factors of Cervical Cancer Progression. Open Med..

[92] Periyasamy M., Singh A.K., Gemma C., Kranjec C., Farzan R., Leach D.A., Navaratnam N., Pálinkás H.L., Vértessy B.G., Fenton T.R. (2017). P53 Controls Expression of the DNA Deaminase APOBEC3B to Limit Its Potential Mutagenic Activity in Cancer Cells. Nucleic Acids Res..

[93] Fenton T.R. (2021). Accumulation of Host Cell Genetic Errors Following High-Risk HPV Infection. Curr. Opin. Virol..

[94] Oh S., Bournique E., Bowen D., Jalili P., Sanchez A., Ward I., Dananberg A., Manjunath L., Tran G.P., Semler B.L. (2021). Genotoxic Stress and Viral Infection Induce Transient Expression of APOBEC3A and Pro-Inflammatory Genes through Two Distinct Pathways. Nat. Commun..

[95] Middlebrooks C.D., Banday A.R., Matsuda K., Udquim K.-I., Onabajo O.O., Paquin A., Figueroa J.D., Zhu B., Koutros S., Kubo M. (2016). Association of Germline Variants in the APOBEC3 Region with Cancer Risk and Enrichment with APOBEC-Signature Mutations in Tumors. Nat. Genet..

[96] Smith N.J., Reddin I., Policelli P., Oh S., Zainal N., Howes E., Jenkins B., Tracy I., Edmond M., Sharpe B. (2024). Differentiation Signals Induce APOBEC3A Expression via GRHL3 in Squamous Epithelia and Squamous Cell Carcinoma. EMBO J..

[97] Chen S., Li X., Qin J., Chen Y., Liu L., Zhang D., Wang M., Wang M., Zhang D. (2015). APOBEC3A Possesses Anticancer and Antiviral Effects by Differential Inhibition of HPV E6 and E7 Expression on Cervical Cancer. Int. J. Clin. Exp. Med..

[98] Cescon D.W., Haibe-Kains B., Mak T.W. (2015). APOBEC3B Expression in Breast Cancer Reflects Cellular Proliferation, While a Deletion Polymorphism Is Associated with Immune Activation. Proc. Natl. Acad. Sci. USA.

[99] Faden D.L., Ding F., Lin Y., Zhai S., Kuo F., Chan T.A., Morris L.G., Ferris R.L. (2019). APOBEC Mutagenesis Is Tightly Linked to the Immune Landscape and Immunotherapy Biomarkers in Head and Neck Squamous Cell Carcinoma. Oral Oncol..

[100] Chen Y.J., Roumeliotis T.I., Chang Y.H., Chen C.T., Han C.L., Lin M.H., Chen H.W., Chang G.C., Chang Y.L., Wu C.T. (2020). Proteogenomics of Non-Smoking Lung Cancer in East Asia Delineates Molecular Signatures of Pathogenesis and Progression. Cell.

